# Smell Dysfunction in Patients with Primary Sjögren’s Syndrome: Impact on Quality of Life

**DOI:** 10.3390/jcm12072724

**Published:** 2023-04-06

**Authors:** Minan Y. Al-Ezzi, Khalid S. Khan, Anwar R. Tappuni

**Affiliations:** 1 Institute of Dentistry, Barts and The London School of Medicine and Dentistry, Queen Mary University of London, London E1 2AD, UK; 2 College of Medicine and Dentistry, Ulster University, Birmingham B4 6BN, UK; 3 Department of Preventive Medicine and Public Health, Faculty of Medicine, University of Granada, 18016 Granada, Spain

**Keywords:** smell, olfaction, quality of life, mucosal dryness, primary Sjögren’s syndrome

## Abstract

Objectives: Patients with primary Sjögren’s syndrome (pSS) often report smell and taste disturbances. However, the correlation between smell impairment and mucosal dryness is not well understood. The objectives of this study were to investigate the following: (1) the prevalence of smell hypofunction in patients with SS; (2) the impact of smell hypofunction on their quality of life (QoL); (3) whether the patients’ smell is correlated with xerostomia; and (4) whether the patients’ smell is affected by taste hypofunction, disease duration, age, smoking or self-reported neuropathy. Methodology: An ethically approved cross-sectional study was conducted on 65 female patients with SS and 62 sex-matched healthy controls. Their smell was assessed using the University of Pennsylvania Smell Identification Test. Their taste acuity was assessed using the Taste Strips Test. A visual analogue scale was used for the self-assessment of smell and taste functions. Xerostomia was assessed by the salivary flow rate, clinical oral dryness score and the Xerostomia Inventory. The patients’ QoL and mental health well-being were assessed using validated questionnaires. Results: In the SS group, the patients’ smell function was impaired in 27/65 patients compared with the controls (15/62, *p* < 0.05), and it did not correlate with the severity of xerostomia, taste acuity (r = 0.05, *p* = 0.6) or self-reported nasal dryness (r = −0.02, *p* = 0.7). In the patients’ group, smell hypofunction was not correlated with disease duration (β = 0.1, 95% CI = −0.07–0.1) or smoking (β = −0.02, 95% CI = −8–7). Age was not correlated with the smell function in the patients’ group (β = −0.1, *p* = 0.5) but was correlated significantly with smell in the healthy participants’ group (β = −0.3, *p* = 0.02). Neuropathy affected 81.2% of the patients’ group. Their QoL and mental health well-being were not affected by smell hypofunction. Conclusion: Smell hypofunction appears to be a clinical manifestation in patients with SS, but it does not seem to be associated with the severity of mucosal dryness or with taste disturbance.

## 1. Introduction

Sjögren’s syndrome (SS) is a chronic autoimmune disease of unknown aetiology that primarily affects the exocrine glands, leading to the functional impairment and dryness of mucosal membranes. Patients diagnosed with SS frequently report dryness of the nasal passages and olfactory disorders, but only a few studies have addressed this problem in these patients.

Smell and taste dysfunctions have been previously reported in patients diagnosed with SS [1,2], but the number of recruited participants was limited in these studies.

However, there are other studies which do not support that smell is significantly more impaired in patients with SS [3,4]. The findings of studies on the aetiology of smell dysfunction have also been contradictory. Some studies have correlated smell disturbance with mucosal dryness of the nasal cavity [1,2]. Others have suggested the systemic inflammatory process in SS as the aetiological factor for smell dysfunction in patients with SS [5], contradicting a previous study in which no association was found between impaired smell function and the inflammatory markers of the syndrome [6].

Peripheral neuropathy is a well-documented symptom in patients with SS [7,8]. Some studies have reported that the integrity of the neurological function of olfaction is important for smell acuity [9,10]. In addition, smell impairment has been recognised as an early feature in patients with neurodegenerative and age-related disorders, such as Alzheimer’s and Parkinson’s disease [11,12,13]. However, whether there is a neurological basis for smell dysfunction in patients with SS has not been studied. In the current study, we investigated whether patients who have peripheral neuropathy also demonstrate impaired smell in an attempt to provide preliminary data for future studies.

It is recognised in the literature that patients diagnosed with SS may have smell and taste problems [14,15], but it is unclear whether smell dysfunction is influenced by taste disturbance in these patients, correlated with the dryness of mucosal membranes or correlated with neuropathy. Therefore, the primary aims of this study were to assess the prevalence of smell dysfunction in a cohort of patients with SS, to evaluate the impact of smell dysfunction on their QoL and to investigate whether the smell function is correlated with the severity of mucosal dryness or with taste dysfunction. The secondary aims were to investigate whether the smell function is correlated with taste, disease duration (the onset of the symptoms), age, smoking or self-reported neuropathy. This study is part of a larger project in which persistent dryness of the mucosal membranes in patients diagnosed with pSS is hypothesised to compromise the senses of smell and taste as well as sexual function, which can affect patients’ quality of life and mental health well-being [16].

## 2. Study Group

The study was based in the Multidisciplinary Sjögren’s Clinic, Institute of Dentistry, Queen Mary University of London, UK. One researcher (MYA) performed the recruitment procedure during the period between 2 March and 30 November 2016, and the investigations were performed in the daytime (10 am–4 pm) for the participants’ convenience. Eligible patients were defined as women diagnosed with pSS according to AECG criterion [17]. Sixty-five patients were recruited from the above clinic or identified by screening 337 patient records on the clinical database. The database was reviewed and suitable patients who had given consent in the past and met the eligibility criteria were sent an invitation pack in the post with full information on the research project. Additionally, the research project was announced on the British Sjögren’s Syndrome Association (BSSA) website, and interested pSS members were invited to contact the research team.

For comparison, healthy participants were recruited from the general population. The project was advertised in the Institute of Dentistry with the contact details of the research team for any interested people to take part. Sixty-two sex-matched healthy individuals aged 18 years or more, who were capable of providing informed consent and who were able to understand verbal and written information in English, with the support of the researcher, were recruited to the control group.

Participants were excluded if they had a current cold/blocked nose, they were pregnant or breastfeeding, and/or they currently or previously had had head and neck radiation, chemotherapy treatments, chronic salivary gland disease or swelling, secondary SS, asthma, allergic sinusitis, uncontrolled diabetes, candidiasis, lichen planus, severe gum disease or dental caries, which can interfere with the taste function and patients’ QoL. A record of the medical history and current medications taken by the study participants was kept to assess whether there was an association with smell or taste function [18]. 

## 3. Methodology

A cross-sectional study was given an ethical approval by a research ethics committee to be carried on 65 primary SS (pSS) female patients diagnosed according to the American European Consensus Group (AECG) criteria and 62 sex-matched healthy volunteers [19]. All study protocols were approved by Research Ethics Committee of London Bridge (Reference number: 15/LO/2064, 10 February 2016) [18]. One investigator performed all assessments in no particular order for both groups. Information on all participants’ oral and general health as well as medications and smoking habits was obtained from their histories and/or medical records.

Smell function was assessed by the University of Pennsylvania Smell Identification Test (UPSIT-40) (Sensonics, Haddon Heights, NJ, USA), which is a forced-choice test for the quantitative assessment of the smell function [20]. The test comprises 40 standardized items distributed into four booklets; each booklet has ten boxes of embedded microencapsulated odours with four different choices provided for each box. Participants had to scratch each box with the provided pencil, sniff the released smell and then select an appropriate match out of the provided options on the relevant page of the booklet. A score was then calculated for the final recognition of each subject. A special version of this test was ordered to match British cultural norms. The smell test results were calculated collectively to indicate acuity of smell in each individual. There was no gradient, and each smell was given a binary value (one of two scores, i.e., yes or no) then added as a total value at the end. A cut-off point for smell dysfunction for all participants was given at ≤30. Taste Strips Test (TST) (Burghart Medical Technologies, Wedel, Germany) was used to assess the threshold of the taste function of four primary tastes: sweet, sour, salty and bitter. These strips were placed on three sites on the anterior 2/3 of the tongue: tip, right side and left side [14]. The taste test results were grouped according to each individual test tested: sweet, sour, salty and bitter [14,18].

Nasal dryness was assessed subjectively by asking patients whether they suffered from this symptom, which is one of the eleven items in the Xerostomia Inventory that is used to assess xerostomia [21]. Xerostomia severity was assessed clinically by stimulated (SSFR) and unstimulated (USSFR) salivary flow rate (SFR) as well as by clinical oral dryness score (CODS) [22,23,24]. World Health Organisation Quality of Life—BRÉF (WHOQOL—BRÉF) and Hospital Anxiety and Depression Scale (HADS) were used to assess their general QoL and mental health well-being [19,25]. A visual analogue scale (VAS) was used for self-rating smell (“How do you rate your sense of smell?’’) by all participants with an arbitrary cut-off value of <50 over a 100-point graded scale. The study group were asked open-ended questions to assess symptoms of neuropathy. These questions are routinely used in neurology clinics at the Royal London Hospital for the clinical assessment of neurological impairment:

- Have you lost feeling in your hands and/or feet?
- Do you have tingling in your hands and/or feet (pins and needles)?
- Do you have numbness in your hands and/or feet?
- Have you suffered from clumsiness?

## 4. Statistical Analysis

Data were analysed using the latest version (version 23) of Statistical Package for Social Sciences, IBM Corporation, SPSS Inc., Chicago, IL, USA statistical software. A pilot study was conducted to help estimate the sample size calculation, which was based on the mean difference of the smell and taste outcomes from a larger study. The power was set at 90% and the level of significance at 5%. It was concluded that a total of 75 participants (patients with SS and healthy volunteers) would be enough to detect that level of difference. The sample was inflated by 20% to give a total of 90 participants (45 patients and 45 healthy volunteers) to account for any potential dropouts. Continuous variables were expressed as mean difference followed by *p*-value or 95% confidence interval (CI). Independent *t*-test, Chi-square test and multiregression analysis test were used. Residual plots were used to assess the quality of regression. Frequency analysis was used to determine the rate of the self-reported neuropathy symptoms by patients.

## 5. Results

Participant recruitment and data collection took place in the period from 2 March to 30 November 2016. Sixty-five patients and sixty-two sex-matched healthy volunteers gave their consent and participated in the study. All were literate with different levels of educational attainment. The age mean (±SD) of the patients was 59 ± 13 (patients’ ages ranged from 24 to 83 years) and the age mean of the healthy volunteers was 43 ± 15 (volunteers’ ages ranged from 21 to 93 years) (Table 1). Smoking was reported in 6% of the participants in each group (*n* = 4 patients, *n* = 4 healthy participants), whilst chewing betel leaves was only reported in 3% of the healthy participants’ group (*n* = 2). In the patients’ group, individuals reported autoimmune diseases; 14% reported arthritis (*n* = 9/65) and 26% reported thyroiditis (*n* = 17/65). The medications reported by the participants were as follows: hydroxychloroquine, pilocarpin, supplements, antidepressants, immunosuppressants, anticoagulants, antihistamines, antihyperthyroids, antihypothyroids, antibiotics, angiotensin, pain relievers, antiacid drugs, hepoglycaemic, inhalers, primary biliary cirrhosis drugs, overactive bladder drugs, topical medicines (eye drops, eye gels, Viscotears (liquid gel), skin creams and Telmesteine) and gabapentine. A multiregression analysis was used to assess the effect of these medicines on the patients group’s smell and taste.

The smell function was statistically significantly impaired in the patients’ group (30 ± 7) compared with the controls (34 ± 5). The mean difference (4, 95% CI = 1.8–6.1) and percentage difference (17.4, *p* = 0.03) of the smell function between the two groups were both statistically significant. Individuals with hyposmia comprised 41.5% (*n* = 27/65) of the patients with SS vs 24.1% (*n* = 15/62) of the healthy controls. In the patients’ self-assessment of smell quality using the VAS, a significant positive correlation was found between the smell function and VAS smell in the patients’ group (r = 0.7, *p* = 0.00). Interestingly, only 7.3% (*n* = 10) of the patients’ group were aware of the loss of their smell acuity (Table 2).

In the patients’ group, xerostomia was found in 78.4% (*n* = 51/65) of the patients using USFR, 64.6% (*n* = 42/65) using SSFR and 84.6% (*n* = 55/64) using CODS assessments (Table 1). The multiregression analysis revealed that the severity of xerostomia assessed by USFR and CODS did not contribute to smell dysfunction in the pSS group (Table 3).

The impairment of the smell function in the SS group was not correlated with their QoL in any of the assessed domains: physical (β = −0.02, 95% CI = −1.4–1.3), mental (β = 0.1, 95% CI = −0.9–1.4), social (β = 0.1, 95% CI = −0.9–1.6) or environmental (β = 0.1, 95% CI = −1–1.3). Additionally, mental health well-being in both domains—anxiety (β = 0.1, 95% CI = −0.2–0.3) and depression (β = −0.1, 95% CI = −0.3–0.2)—was not associated with smell dysfunction (Table 4).

### Smell and Taste

When the data of both groups were pooled (total population *n* = 127), a significant positive correlation was found between the smell and taste functions (r = 0.3, *p* = 0.05). However, the smell function was not a good predictor of taste acuity in the total population of the study (β = 0.09, 95% CI = −0.03–0.1).

**Figure 1:**
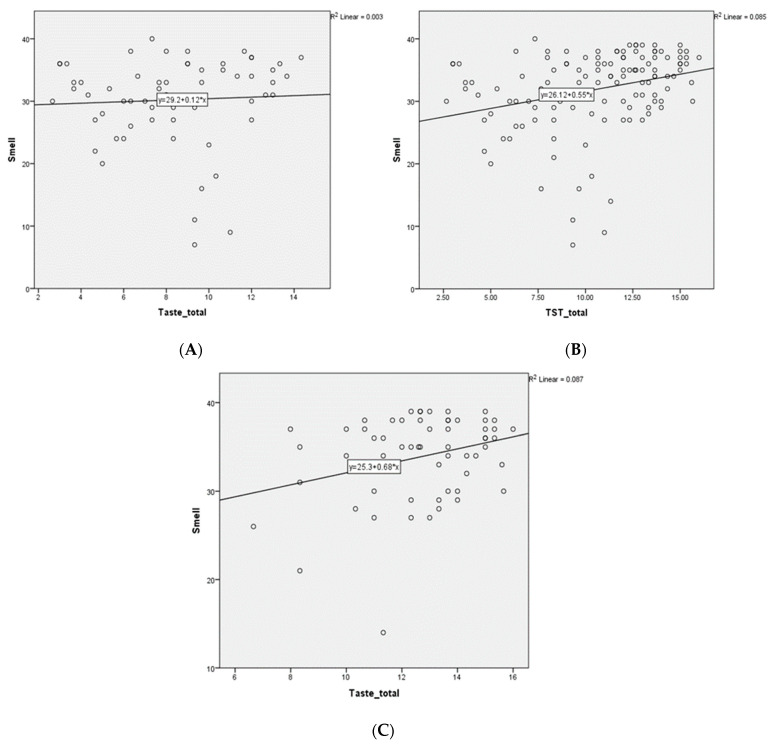
Correlation between smell and taste functions in the study. (**A**) Patients’ group; (**B**) Healthy participants’ group; (**C**) Total population of the study (patients and controls).

In the SS group, no significant correlation was found between the smell and taste functions (r = 0.05, *p* = 0.6), unlike in the healthy group, in which a significant positive correlation was established between smell and taste (r = 0.2, *p* = 0.05). In the regression model, the smell function was not a predictor of taste acuity in the SS (β = −0.06, 95% CI = −0.1–0.08) or in the control groups (β = 0.2, 95% CI = −0.03–0.2) (Figure 1).

No significant correlation was found between self-reported nasal dryness, which was assessed by one of the Xerostomia Inventory items, and the smell function (r = −0.02, *p* = 0.2) in the study group. Similarly, the smell function in the patients’ group was not correlated with disease duration (β = 0.1, 95% CI = −0.07–0.1) or smoking (β = −0.02, 95% CI = −8–7). Age did not seem to influence the smell function of the patients’ group (β = −0.1, *p* = 0.5), but there was a significant negative correlation between age and smell in the healthy participants’ group (β = −0.3, *p* = 0.02).

## 6. Effect of Medicines on the Smell Function and Mucous Membrane

In the patients’ group, topical medicines were correlated with the smell function and were a good predictor of its impairment (β = −0.4, 95% CI = −9–−0.7) and of the severity of xerostomia (β = 0.4, 95% CI = 0.3–2.2) assessed by CODS. Nasal dryness, which was assessed by item 11 of the Xerostomia Inventory, correlated significantly with hydroxychloroquine (β = 0.4, 95% CI = 0–2.1) and supplements (β = −0.3, 95% CI = 0–−2). Pain relief (Aspirin, Codeine, Co-codamol, Diclofenac, Fentanyl, Naproxen, painkillers, Paracetamol) were significantly associated with the impairment of the taste function (β = 0.3, 95% CI = 0.1–3.8). Gabapentin (β = −0.2 95% CI = −6.4–0.3) and inhalers (β = −0.2. 95% CI = −8.8–1.2) were correlated with the taste function but not significantly.

## 7. Discussion

This study was designed to assess the smell function in patients diagnosed with SS and its correlation with the dryness of the mucous membrane. We investigated the effect of smell impairment on the QoL and mental health well-being of patients with a confirmed diagnosis of SS. Our results demonstrated that patients with SS are more likely to have smell dysfunction compared with healthy controls. The dryness of the mucosal membranes was not the key indicative factor for smell impairment, as was previously suggested in the literature [1,2].

In this study, the statistically significant mean difference in the smell function between both groups was small. In the SS group, 41.5% exhibited disturbances in smell function compared to the healthy volunteers (24%), which may indicate that SS can cause smell dysfunction. It is worth noting that the overall prevalence of olfactory dysfunction in the general population, which was reported in a recently published systematic review and meta-analysis (22.2%), is comparable with our findings of smell dysfunction in the control group (24.1%) [26].

Interestingly, the majority of the patients with SS who had abnormal UPSIT results were unaware of any smell problems and reported no change in their smell function or acuity. Perhaps this is attributed to coping mechanisms that patients with SS develop over time.

Henkin et al. [1] and Kamel et al. [2] suggested a correlation between the deterioration of smell function and the dryness of the nasal mucosa in patients with SS. However, our results did not support the findings of these studies and revealed no correlation between smell dysfunction and the dryness of the mucosal linings in SS patients. Our findings, however, are in line with those of a study by Rasmussen et al. [3], which demonstrated that the smell threshold is not associated with the severity of the dryness of the nasal mucosa in patients diagnosed with SS.

Due to the rarity of patients diagnosed with SS, it was difficult to exclude those on medications, which would otherwise limit the pool of patients required for the current investigations. When confidence intervals are reported, our interpretation is aided by the knowledge of range of possible results rather than a single *p*-value. Therefore, a Bonferroni correction is recommended for consideration in the future.

Age was not correlated with smell in our SS group, which contradicts a previous statement on the negative correlation between age and smell in patients with SS [2]. However, in our healthy group, there was a significant negative correlation between age and smell, which is an anticipated regression of the smell function with age [20,27,28].

Smoking had a weak association with the smell function in the patients’ and healthy volunteers’ groups. This finding supports previous evidence which demonstrated an association between heavy smoking and smell deficits [2,29,30]. Interestingly, our data showed that the highest score on the smell test (39/40) was recorded by a healthy participant who reported smoking 20 cigarettes per day and was considered to be a heavy smoker. This participant reported that their smell acuity had not changed, which may be due to the continuous renewing process of the nasoepithelium due to the exposure to smoke particles.

The disease duration did not influence the smell function in the patients group. This was a surprising finding, as it was anticipated that with time, the disease would progress and therefore patients with a longer disease duration would be more likely to be symptomatic. We are unaware of any studies that have investigated the association of disease duration with the smell function in patients diagnosed with SS; therefore, studies for comparison are not applicable.

Within the SS group, patients who were on topical medicines (e.g., eye drops, eye gels, skin creams) had significantly more smell disturbances (β = −0.4, 95% CI = −8.6–−0.7) than those who did not report using topical preparations. It is presumed that patients with more severe SS symptoms are the ones who are more likely to have smell dysfunction and more likely to be on topical medications.

### Correlation between Smell and Taste 

The evidence in the literature is conflicting on whether a correlation between smell and taste exists. Our study revealed that smell and taste functions were correlated in the study population as a whole (*n* = 127) and in the control group (*n* = 62) but not in the patients’ group (*n* = 65). This can be attributed to the presence of underlying factors that impeded the correlation of both variables in the SS group. In the current study, the prevalence of neuropathy was 81.2% in our pSS population. The symptoms that were reported ranged from “lost feeling” to “tingling”, “numbness” or “clumsiness”. Our results support previous findings of smell disturbances in patients with polyneuropathic symptoms, in which neurological function integrity was found to be important for olfaction acuity [9,10]. The evidence in the literature supports the fact that Sjögren’s syndrome can cause minor cognitive dysfunction associated with the duration and severity of the symptoms of the disease, without manifestations of mental disorders or even central nervous system involvement [31]. This could be attributed to bioelectrical dysfunction in an ongoing long-term autoimmune process that can affect other sensory systems, such as the auditory pathway [32]. Therefore, neuropathy should be considered as a possible factor compromising the smell function in patients diagnosed with SS. However, assessing the nerve function of the smell sensation was beyond the remit of the present study. Furthermore, factors such as mucosal oedema or nasal crusting may potentially be possible contributing factors that compromise smell in patients with SS. The data in this preliminary study suggest that including CT scans and endoscopy in studies investigating smell disturbance would lead to more robust results and stronger evidence for the aetiology of olfactory dysfunction.

We concluded that smell dysfunction did not compromise the QoL or the mental health well-being in patients diagnosed with SS. This finding was similar to our findings reported in a previous publication showing that taste impairment in patients with SS did not compromise their QoL or mental health well-being [14]. These findings indicate that the smell and taste problems were not identified as significant health issues by patients with SS. Our results contradicted those of others, who have suggested that the impairment of smell and taste contributes to a diminished QoL in patients with SS [2].

## 8. Conclusions

Irrespective of age, the smell function was affected in patients diagnosed with SS, but it was not influenced by dryness of the mucosal linings, neuropathy or taste. It appears that these patients can cope with reduced smell function without any impact on their QoL and mental health well-being [18].

## Figures and Tables

**Table 1:** Characteristics of patients and healthy volunteers.

Characteristics	Patients *n* = 65	Volunteers *n* = 62
Age	Mean ± SD 59 ± 13	Mean ± SD 43 ± 15
Smoking	2 ± 0.2	4 ± 1
Smokeless tobacco	1 ± 1.4	2 ± 0.1
Alcohol	1.3 ± 1.2	1.6 ± 0.5
Mouthwash	1.2 ± 1	1.5 ± 0.5
USFR *	0.13 ± 0.1	0.6 ± 0.4
SSFR *	0.6 ± 0.7	11.8 ± 26
CODS *	16 ± 30	0.7 ± 1
Xerostomia Inventory	48 ± 5	16.6 ± 5
VAS * smell	7.5 ± 17	11.2 ± 25
Disease duration	17.2 ± 16	---

* USFR: unstimulated salivary flow rate; cut-off value is ≤1.5 mL of saliva in 15 min. * SSFR: stimulated salivary flow rate; cut-off value is ≤0.6 mL/min of the whole stimulated salivary flow rate. * CODS: clinical oral dryness scale. Mild dryness is indicated with scores ranging from one to three. Moderate dryness refers to scores ranging from four to six and severe dryness for scores from seven to ten. * VAS: visual analogue score; cut-off value was specified at <50, indicating a poor rating.

**Table 2:** Comparison of the dysfunction rate of the study outcomes in pSS patients compared with healthy volunteers.

Test	pSS Group Mean Age: 59 95% CI = 59–62	Healthy Volunteers’ Group Mean Age: 43 95% CI = 39–47	Mean Difference (95% CI)	*p*-Value	Type of Test
Smell function ^1^	41.5% (*n* = 27/65)	24.1% (*n* = 15/62)	3.9 (1.8–6)	<0.05	Clinical
Taste function ^2^	54% (*n* = 34/63)	8.3% (*n* = 5/60)	4.3 (3.4–5.2)	<0.05	Clinical
Quality of life ^3^ Psychological domain (D2)	47.7% (*n* = 31/65)	9.8% (*n* = 6/61)	11.8 (6.9–16.6)	<0.05	Questionnaire
Quality of life ^3^ Social domain (D3)	44.6% (*n* = 29/65)	21.3% (*n* = 13/61)	11.9 (5.2–18.7)	<0.05	Questionnaire
Quality of life ^3^ Environmental domain (D4)	21.5% (*n* = 14/65)	9.8% (*n* = 6/61)	6 (0.9–11.2)	<0.05	Questionnaire
Mental health well-being ^4^ Anxiety	58.5% (*n* = 38/65)	21% (*n* = 13/61)	2.8 (1.5–4)	<0.05	Questionnaire
Mental health well-being ^4^ Depression	32.3% (*n* = 21/65)	8.2% (*n* = 5/61)	3.5 (2.3–4.6)	<0.05	Questionnaire

^1^: Calculated with normal smell function as ≥30 on a scale from 0 to 40; ^2^ normal taste function as ≥9 on a scale from 0 to 16; ^3^ overall QoL as ≥ 60 on a scale from 0 to 100; ^4^ and normal HADS score as <8.

**Table 3:** Coefficients table for the multiregression analysis of the impact of xerostomia assessed by USFR, SSFR and CODS on the smell function of the pSS group.

Xerostomia Tests	Standardized Coefficients	95% CI for B	R^2^
USFR	0.03	−11.2–15	0.19
SSFR	0.1	−1.6–4	0.2
CODS	−0.1	−1.1–0.8	0.19

**Table 4:** Coefficients table for the impact of smell dysfunction on QoL and mental health well-being of the pSS group.

	Standardized Coefficients (B)	95% CI for B	R^2^
Physical domain (WHOQoL-BRÉF)	−0.018	−1.4–1.3	0.35
Psychological domain (WHOQoL-BRÉF)	0.1	−0.9–1.4	0.31
Social domain (WHOQoL-BRÉF)	0.1	−0.9–1.6	0.51
Environmental domain (WHOQoL-BRÉF)	0.06	−1–1.3	0.31
Anxiety (HADS)	0.1	−0.3–0.4	0.39
Depression (HADS)	−0.1	−0.4–0.2	0.47

## Data Availability

The datasets used and/or analysed during the current study are available from the corresponding author on reasonable request.
